# A comparative analysis of perennial grass–legume mixtures for biomethane production

**DOI:** 10.1016/j.heliyon.2024.e33401

**Published:** 2024-06-21

**Authors:** Rita Bužinskienė

**Affiliations:** a Department of Applied Economics, Finance and Accounting, Faculty of Bioeconomy Development, Vytautas Magnus University Agriculture Academy, Studentu Str. 11, Akademija, LT-52261, Kaunas Dist., Lithuania; b Department of Business and Accounting, Faculty of Business and Technology, Šiauliai State Higher Education Institution, Aušros ave. 40, LT-76241, Šiauliai, Lithuania

**Keywords:** Biomethane natural gas, Agriculture development, Perennial bell and legume grasses, Comparative analysis

## Abstract

Examining the case of Lithuania, this study comparatively analyzed five perennial grass–legume mixtures in terms of biomethane production. Every mixture was divided into two parts: long (during the fifth year or beyond) and short (during the first four years) time periods. The analysis includes three types of perennial bell grass: Timothy, P. Ryegrass, C. Cocksfoot, and one legume grass Red clover. With this study, we aimed to evaluate how perennial grass–legume mixtures can promote biomethane uptake in Lithuania. Through analyzing the efficiency and consequences of government subsidy measures, this study aimed to address the question of how governmental assistance can promote the growth of the biomethane industry, specifically focusing on the utilization of perennial grass–legume mixtures. This study used seven financial indicators, including subsiding policy, in order to gain a deeper understanding of mixtures for biomethane production. The analysis revealed that the best mixtures for biomethane production with subsidies were the second (Red clover 35 % + Timothy 45 % + Ryegrass 20 % grass mixture) and fourth scenarios (Red clover 55 % + Ryegrass 45 % grass mixture). The first (Red clover 35 %. + Timothy 25 % + Ryegrass 20 % + Cocksfoot 20 % grass mixture), third (Red clover 55 % + Timothy 45 % grass mixture), and fifth scenarios (Red clover 55 % + Cocksfoot 45 % grass mixture) had the smallest positive effects. The results showed that, in Lithuania, in order to encourage farmers to produce biomethane, subsidy policies are needed. Incentives for engaging with this activity are necessary, as the income earned does not cover the costs incurred; unfortunately, biomethane production is unprofitable without subsidy. As such, our recommendation is to develop a long-term subsidy policy to promote biomethane production, focusing on the effectiveness, particularly in the Lithuanian context, of utilizing mixtures of perennial grasses. Further research and policy interventions are needed to address the opportunities associated with scaling synergy between perennial energy cops and environmental sustainability in bioenergy crop cultivation.

## 1 Introduction

The European Commission's recognition of biomethane production as a key component of the renewable natural gas market underscores its growing importance in achieving sustainable energy targets. As the REPowerEU plan outlines, the Commission has set ambitious goals, including reaching a target of 35 billion cubic meters (bcm) of renewable natural gas production by 2030 [1]. The possibilities of biomethane production are expansive, with potential uses from industry to the transport sector. The latter in particular depends on the use of fossil fuels. The expansion of biomethane production processes offers tangible benefits in terms of increasing the utilization of renewable energy sources, strengthening energy independence, and promoting sustainable resource management practices. Positioned as a renewable natural gas substitute, biomethane is derived from biogas, which is produced through the anaerobic digestion of organic matter in an oxygen-free environment. This process not only produces a clean and sustainable energy source but also involves the removal of CO₂ and other contaminants from biogas, enhancing its environmental credentials. By volume, the methane content of biogas can range from 45 % to 75 %, with the biggest remaining portion being CO₂. This means that the energy content of biogas can vary; the lower heating value (LHV) is between 16 MJ per cubic meter (MJ/m₃) and 28 MJ/m₃ (WEOSP, 2020). In biogas plants, the biomethane production process consists of six stages: (1) raw materials are transported, stored, and fed into a bioreactor; (2) biogas production is carried out in bioreactors (for up to 60 days); (3) biogas is stored; (4) biogas for heat generation is used as an external heat source; (5) biomethane is purified (the majority of biogas is methane (CH₄) (55–70 %.), carbon dioxide (CO₂) (27–44 %.), hydrogen sulfide (H₂S) (up to 3 %), and water vapor (H₂) (up to 1 %); only trace amounts of other gases are observed); and (6) the obtained substrate (digestate) is considered a high-quality fertilizer [2].

Lithuania does not yet produce its own biomethane; it is imported from other countries through international certificates. This reliance can affect energy security and economic stability. Lithuanian farmers have faced difficulties in maintaining or expanding their perennial grassland areas due to competing land uses. These areas are vital for livestock grazing, soil conservation, and biodiversity maintenance. As noted in 2023, the significant decrease in the area of perennial meadows in Lithuania (by more than 5 %) indeed poses a serious threat to direct payments to farmers through European funds [3].

The growing demand for biomethane could present an opportunity to address the decline of meadow cultivation in Lithuania. Encouraging the cultivation of perennial grass–legume mixtures for biomethane production could indeed be highly beneficial for farmers. Germany is a world leader in the use of permanent grasslands for biogas production (around 2 bcm) [4], especially in terms of integrating forage legumes for bioenergy production into cropping systems without any restrictions for food and feed production [5]. With the exception of Germany, permanent grassland is still not considered the main raw material for biogas production in Europe, as it is thought to only be appropriate for feeding animals. If there is a shift towards lower levels of animal husbandry in Europe in the future, the land that is currently used for grazing or forage production could be freed up, thus creating opportunities for repurposing some of these permanent grassland areas for biomethane production.

However, while biomethane holds promise, its widespread adoption faces challenges, such as in ensuring the availability of sustainable feedstock sources to support biomethane production. The choice of grass type and the different ways to utilize the capabilities of biogas plants must be assessed in advance. Biogas plants that only use crops such as rapeseed, wheat, rye, and ryegrass are sensitive to changes in the raw material market [6]. Amaleviciute-Volunge et al. (2020) [7] affirmed that achieving the highest methane yield is contingent upon the selection of appropriate grass species. Specifically, they identified certain grass species—such as C. Sainfoin (Onobrychis viciifolia), C. Cocksfoot (Dactylis glomerata), and P. Ryegrass (Lolium perenne)—as particularly conducive to biomethane production. Iliev et al. (2020) [8] underscore how the primary determinants of biogas yield are the types of feedstocks employed and their duration in the reactor. This highlights the significance of selecting appropriate feedstocks and optimizing the retention time within anaerobic digestion systems to maximize biogas production efficiency. Tomić et al. (2007) [9] revealed that legume–grass mixtures can thrive in various soil types, showcasing adaptability across different agricultural settings. The growth conditions required for these mixtures are not overly complex, making them accessible and manageable for farmers. Additionally, this study highlighted the significant potential yield of dry matter (DM): up to 20 tons per hectare. Alberici et al. (2022) [4] claim that permanent grassland has the potential to be used as a feedstock for biogas use in the future. Until recently, permanent grassland was considered the main feedstock for feeding animals. However, in the future, if there are lower amounts of animal husbandry in Europe, more grassland areas will be available for use in biogas production (around 2 bcm). Selecting optimal grasses with favorable biochemical properties can also enhance biogas yield and efficiency. Some researchers disclosed that, in their dry matter content, perennial grasses typically contain 25–40 % cellulose, 15–50 % hemicellulose, and 10–30 % lignin [10]. Characterized by their relatively high cellulose and hemicellulose contents and lower lignin content, perennial grasses are well-suited for biomethane production through anaerobic digestion. During the biogas production process, lignin degradation tends to be slower and less efficient compared to that of cellulose and hemicellulose [11]. To ensure optimal biogas yield, it is often recommended that the lignin content be kept below 15 % of the dry matter content [12]. Reduced methane yields are linked to elevated lignin and reduced hemicellulose content [13].

As society increasingly relies on biomethane, it is crucial to expand our focus beyond single-species cultivation. Several studies have highlighted the potential of perennial plant mixtures in meeting this demand. For instance, Kintl et al., 2020 [14] examined a mixture of maize and white sweet clover, while Cossel et al., 2021 [13] explored perennial wild plant species like common tansy, brown knapweed, mugworts, cup plant, Virginia mallow, and maize. Additionally, Kupryś-Caruk et al. (2023) [15] investigated Miscanthus, cordgrass, switchgrass, and big bluestem, while Oleszek et al., 2020 [16] studied maize, sorghum, sunflower, triticale, reed canary grass, and Virginia mallow. Notably, Sida hermaphrodita [17] and grass cocksfoot [18] have also been examined for their suitability as regenerative feedstock for bioenergy production.

This study aims to evaluate how a mixture of perennial grasses and legumes can promote biomethane uptake in Lithuania. Utilizing a comparative analysis approach, this study seeks to identify the most effective value chain for biomethane production using perennial grasses and legumes. To address the existing research gaps, this study makes a significant contribution to the literature on biomethane production by focusing in particular on optimizing feedstock utilization efficiency. This aspect is crucial as it directly impacts biomethane production systems' overall sustainability and economic viability.

The novelty of this research lies in exploring the potential of biomethane as a renewable energy source using various perennial grass–legume mixtures. This study focuses on Lithuania's approach, which is particularly relevant, given the current context, to identifying suitable perennial grass–legume mixtures for biomethane production and, indeed, pertinent to global efforts in sustainable energy solutions. Our comparative analysis includes the currently implemented subsidy policies in Lithuania; by analyzing the effectiveness and impact of these subsidy policies, our research seeks insights into how government support can facilitate the growth of the biomethane sector and accelerate Lithuania's transition to a more sustainable energy future. The results indicate that, for biomethane production to be economically viable, all perennial grass–legume mixtures require subsidizing. Without subsidies, the production costs outweigh the revenue generated, and a positive net cash flow may be achieved only by the third year.

## 2 Materials and methods

The study investigated the efficacy of four distinct perennial grass–legume mixtures, with Red Clover (Trifolium pratense) standing out as one of the primary legumes. These mixtures combined Red Clover with other notable bell grasses, including Timothy (Phleum pratense), P. Ryegrass (Lolium perenne), and C. Cocksfoot (Dactylis glomerata). These species exhibit characteristics that contribute to optimal methane yields during anaerobic digestion processes. The mixtures of legumes and bell plants do not directly compete with each other for water, mineral nutrients, and light. Beyond their economic significance, these plants play crucial roles in the ecological and biological processes of meadows, contributing to their sustainability and biodiversity. Their growth not only generates abundant biomass suitable for biomethane applications but also enhances soil structure and fertility [19]. Characteristic values of perennial grass–legume mixtures for biomethane production are shown in Table 1 [7,20,21].

**Table 1:** Characteristics of perennial grass–legume mixtures for biomethane production.

Characteristics	Unit	Red clover	Timothy	Perennial Ryegrass	Common Cocksfoot
Methane yield	m³ CH₄-yr/ha	1350–5985	1362-5800	2500–6150	1480–3800
Biogas yield	m³/ha	2455–10882	2476–10546	4546–11182	2691–6909
Fresh matter (FM) yield	t/ha	15–58	27–55	27–46	24–30
Dry mater (DM) yield	m³/t	5–19	9–18	9–15	8–10
Methane DM yield	CH₄ m³/t	270–315	151–322	270–410	185–380

Red Clover (Trifolium pratense) is a traditional legume grass widely cultivated as a forage grass in Lithuania. Typically mixed and sown with bell grasses, it has a growth cycle of around 3 years. Red clover thrives in soil with a pH range of 6–7, although it is known to be somewhat demanding regarding soil quality [22]. Timothy (Phleum pratense), a perennial grass belonging to the bellflower family, is another important forage grass in Lithuania. This grass is often cultivated as an important fodder plant, but is somewhat rarer in the northern part of Lithuania, thriving in heavier, moist soil. It grows luxuriantly in meadows, pastures, or along riversides. Common cocksfoot (Dactylis glomerata) is a forage grass belonging to the Poaceae family, known for its rich nutrient content and abundant foliage. It thrives in diverse habitats including meadows, forest clearings, and woodlands. This grass species prefers fertile soil with moderate humidity levels, where it exhibits vigorous growth and lush foliage. Perennial ryegrass (Lolium perenne), a member of the Poaceae family, holds significant agricultural importance in Lithuania and is regarded as one of the most valuable plant species. It thrives in various habitats including meadows, pastures, and along roadsides. However, it is particularly demanding and requires soil with specific qualities. Perennial ryegrass flourishes in fertile, well-drained clay and loam soils, while it tends to struggle in acidic soil conditions [23].

This comparative analysis includes five perennial grass–legume mixtures that could be advantageous for biomethane production. The five different scenarios were compiled taking into account the additional information provided by Butkevičienė (2018) [24]. In each scenario, the allocation of percentages to perennial grass–legume mixtures varied, with the composition predominantly consisting of clovers and bell grasses. For short periods, the mixture typically comprises 50–60 % clovers and 40–50 % bell grasses. In contrast, for longer periods, the distribution shifts towards 25–35 % clovers and 65–75 % bell grasses. Hence, every scenario is divided into two parts: perennial grass–legume mixtures, which can be used for about 5 or more years (*long-term grasslands – 1–2 scenarios* (Table 2)), and perennial grass–legume mixtures, which can be used for up to 4 years (*short-term grasslands – 3–5 scenarios* (Table 3)). In these scenarios, the mixtures combine and balance the benefits of clovers (nitrogen fixation, soil improvement, biodiversity support) and bell grasses (rapid growth, soil stabilization, long-term ground cover). By adjusting the percentages based on the time frame and specific objectives, these mixtures can be tailored to suit different agricultural or ecological needs.

**Table 2:** Long-term perennial grass–legume mixtures.

Scenarios	Red clover 35 %. + Timothy 25 % + Ryegrass 20 % + Cocksfoot 20 % grass mixture *(Scenario 1)*	Red clover 35 % + Timothy 45 % + Ryegrass 20 % grass mixture *(Scenario 2)*
Methane, m³CH₄/ha	3572	3760
Biogas yield m³/ha	6495	6837
Dry matter yield, t/ha	12	13
Methane m₃/t DM	287	278
Fresh matter yield, t/ha	36	39
Grass required yield, t/ha	32006	32787
Agricultural land, ha	896	851

**Table 3:** Short-term perennial grass–legume mixtures.

Scenarios	*Red clover 55 % + Timothy 45 % grass mixture (Scenario 3)*	Red clover 55 % + Ryegrass *45 % grass mixture (Scenario 4*	*Red clover 55 % + Cocksfoot 45 % grass mixture (Scenario 5)*
Methane, m³CH₄/ha	3629	3963	3205
Biogas yield m³/ha	6582	7206	5828
Dry matter yield, t/ha	13	12	11
Methane m₃/t DM	267	316	288
Fresh matter yield, t/ha	39	37	32
Grass required yield, t/ha	33975	29470	32173
Agricultural land, ha	882	807	998

Long-term perennial grass–legume mixtures have the capacity to persist for 5 years or more. These mixtures typically include Red clover, a legume grass, along with various bell grasses such as timothy, common cocksfoot, and perennial ryegrass. They thrive in slightly acidic soil, with a pH of approximately 5.8, that is devoid of carbonates and with groundwater at a depth of 1.3 m [25,26].

Data on the short-term perennial grass–legume mixtures are shown in Table 3.

Short-term perennial grass–legume mixtures are typically maintained for a cycle of 4 crop years. The primary grass in these mixtures is Red clover, a legume grass that is commonly paired with components such as timothy and perennial ryegrass. In lighter soils, Red clover can be combined with common cocksfoot. An essential requirement is that leguminous grasses constitute the majority, ranging from 55 to 80 percent. This proportion ensures an adequate supply of biological nitrogen for bell grasses [25,26].

Here, the biomethane production process is analyzed in an existing biogas plant with a capacity of up to 400 CH₄-m³/h. During the first year, 1000 thousand m³ of biomethane will be produced. In the second year and beyond, it will reach the maximum production capacity of 2085 thousand m³ of biomethane per year [2]. Biomethane will be injected into the natural gas network. The analytical data are shown in Table 4.

**Table 4:** Characteristics of a biogas plant for biomethane production.

Characteristics	Unit	Quantity	Assumptions
Biogas plant capacity	CH₄-m³/h	400	
Capacity of biomethane production	thousand m³/yr.	1000	1 year
		2085	2 years and above
Biomethane	kWh	10 600	1 year
		22 104	2 years and above
Digestate	50 m³/ha		

Note: Biomethane conversion 1 m³ ≈ 10.6 kWh^1^ [27]; digestate volume of 85 mL, equal to 50 m³/ha^2^ [28,29].

The values of biomethane production, digestate, and subsidy are shown in Table 5.

**Table 5:** Economic data on biomethane production, digestate, and subsidy.

Components	Unit	Value
Biomethane purchase price^a^	EUR/kWh	0.09
Digestate purchase price^a^	EUR/m³	6.4
Subsidy	thousand EUR	4000

Note.

a It is assumed that the prices of biomethane and digestate would grow by 10 % each year.

The accepted purchase price of biomethane is EUR 0.09/kWh [8,30], while that of digestate is EUR 6.4/m³ [31]. The subsidy is intended to help in the development of biomethane gas production facilities [32], and data on the main investment areas for these kinds of facilities are shown in Table 6.

**Table 6:** Investment data for biomethane production equipment.

Components	Amount, thousand EUR
Substrate storage, mobile equipment, and technological appliances, initial costs	920
Digester (fermenter) production	682
Gas utilization and control (including biogas processing and feeding) production	1954
Digestate storage	433
Planning, approval, and commissioning	282
Property costs, property development, road and parth construction	438
Biomethane upgrade unit	1320
***Total:***	***6029***

The primary determinants of biogas yield are the types of feedstocks utilized as input, with less emphasis placed on the specific anaerobic digestion technology employed. Additionally, the length of time the feedstocks spend in the reactor significantly influences biogas production. Therefore, it is crucial to assess the energy potential of the selected substrate mix before use [8]. The economic values of technology operations for grass–legume mixtures are presented in Table 7 [[33], [34], [35]].

**Table 7:** Values of technology operations for perennial grass–legume mixtures.

Components	Value^a^
Plowing Cultivation	77 EUR/ha 46 EUR/ha
Sowing	60 EUR/ha
Rolling	20 EUR/ha
Fertilization 2 time per year	9 EUR/ha
Herbicides 2 time per year	12 EUR/ha
Harvesting 2 time per year	43 EUR/ha
Crop shredding	111 EUR/ha
Grass collection and loading	33 EUR/ha
Transportation	3 EUR/km
Ensilage^c^	6.33 EUR/t
Indirect costs^b^	10 % per direct cost
Biogas plant operations	12 % per investment capital

Notes.

a These values were taken at an average price. It assumed that the price of technology operations (except biogas plant operations) would grow by 3 % each year.

b Indirect costs include equipment and machinery depreciation, workers' salaries, and other indirect costs.

c Green biomass is transported by heavy vehicles from farmers and agricultural companies; it is ensiled by a tractor (335 kW) and later transferred to the bioreactor.

Our comparative analysis is based on the following assumptions: (1) all grass–legume mixtures have the same growing conditions; (2) all field cultivation activities are outsourced; (3) the land for growing grass–legume mixtures is owned by farmers and agriculture companies; (4) indirect costs make up 10 % of direct costs; and (5) the ensiling process begins when grass biomass is delivered to the biogas plant at a rate of 60 tons per hour. It must be pressed by a 15-tonne tractor at a driving speed of 4 km per hour [36]. Finally, (6) the biogas plant incurs annual costs of no more than 12 % of the capital invested for biomethane to be installed [35].

A quantitative method approach was applied to evaluate the biomethane produced from a perennial grass–legume mixture. This kind of method helps us compare options and make useful, informed decisions. It takes the comparative evaluation with subsidies and without subsidies approach. In this study, our comparative evaluation of approaches, both with subsidies and without, uses the interval and relation scale of measurements, as expressed by quantitative indicators [[37], [38], [39]]:

***Return of investment (***ROI***)*** – is calculated as an investor's net income divided by the investment's total cost. The indicator represents the profitability metric used to evaluate how much an investment has performed, Equation (1):

(1) $$ROI=\frac{NI}{CI}$$

where,

NI− Net income (total revenues minus cost of investment), EUR;

CI− Cost of investment, EUR.

***Profit margin ratio***(PM) is calculated as the current year's profit, divided by the total revenue of the current year. It represents the sales profitability percentage that has turned into profits, Equation (2):

(2) $$PM=\frac{\left(INC-DC-IC-BP\right)}{INC}$$

where,

INC− Income of biomethane production, EUR;

DC− direct cultivation expenses, EUR;

IC− indirect cultivation expenses, EUR;

BP− biogas processing expenses, EUR;

***The cost-price of produced biomethane***(BC) is calculated as the cost of the current year, divided by the quantity of output produced. This represents the costs of production referring to all the expenses incurred in the process of bioethanol production. It can create opportunities for higher profitability, Equation (3):

(3) $$BC=\frac{\left(DC+IC+BP\right)}{Q}$$

where,

Q− quantity of output (tons).

***Financial net present value*** (FNPV) represents, how the cash flow for the relevant year covers investments; Equation (4):

(4) $$FNPV=\frac{{NCF}_{0}}{{\left(1+d\right)}^{0}}+\frac{{NCF}_{1}}{({1+d}^{)1}}+\ldots \frac{{NCF}_{t}}{{\left(1+d\right)}^{t}}$$

where,

${NCF}_{0,1\ldots ..,t}\;-$ cash flow for the relevant year (EUR);

d− discount rate of 5 % was used for all calculations (r = 5);

t− time period (years).

If FNPV (I) < 0, the net income flows do not cover the investment, so the financial benefit will not be obtained. If FNPV (I) > 0, the net income flows cover the investment, so the project is financially attractive to investors.

After net present value analysis is completely important to examine the internal rate of return (IRR). It is one of the methods that can compare investment on scenario yield. The scenario with the biggest internal rate of return is usually preferred.

***Financial benefit-cost ratio***, FBCR – the indicator represents, how many times the financial benefits generated by the investment project exceed the financial costs required for its implementation, Equations (5), (6), (7), (8), (9):

(5) $$FBCR=\frac{OINCF}{INCF-RVNCF+OENCF}$$

(6) $$OINCF={OINCF}_{0}*\frac{1}{{\left(1+d\right)}^{0}}+{OINCF}_{1}*\frac{1}{({1+d}^{)1}}+\ldots {OINCF}_{t}*\frac{1}{{\left(1+d\right)}^{t}}$$

(7) $$INCF={INCF}_{0}*\frac{1}{{\left(1+d\right)}^{0}}+{INCF}_{1}*\;\frac{1}{({1+d}^{)1}}+\ldots {INCF}_{t}*\frac{1}{{\left(1+d\right)}^{t}}$$

(8) $$RVNCF={RVNCF}_{n}*\frac{1}{{\left(1+d\right)}^{n}}$$

(9) $$OENCF={OENCF}_{0}*\frac{1}{{\left(1+d\right)}^{0}}+{OENCF}_{1}*\;\frac{1}{({1+d}^{)1}}+\ldots {OENCF}_{t}*\frac{1}{{\left(1+d\right)}^{t}}$$

where,

OINCF− operating income net cash flow for year t, EUR;

INCF− investment net cash flow for year t, EUR;

RVNCF− the residual value net cash flow for last year t, EUR;

OENCF− operating expenses net cash flow for year t, EUR;

*n*— project last reporting period (years).

If the scenario has a financial benefit-cost ratio greater than 1.0, the project delivers a positive net present value to investors. If the scenario has a financial benefit-cost ratio less than 1.0, the project delivers a negative net present value to investors.

The ***Net cash flow*** is calculated as the difference between positive and negative cash flows in each year of the reported period. Every year, the net cash flow is carried forward to the next year. This accumulated net cash flow shows whether, during the reported period, the expected revenue will cover the costs in the relevant year. The project promoter must assess the funding needs and anticipate funding sources to meet them.

## 3 Results and discussion

**Fig. 1:**
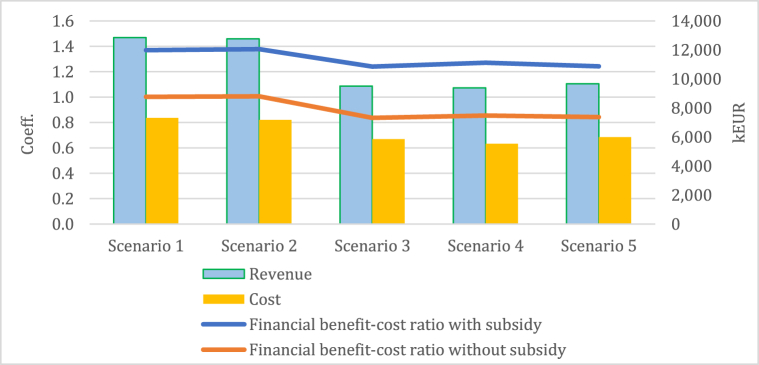
The financial benefit–cost ratio analysis of perennial grass–legume mixtures.

Fig. 1 presents a financial benefit–cost ratio analysis of five perennial grass–legume mixtures in Lithuania. The results consist of two positions: with and without subsidy. Also, scenarios 1 and 2 depend on the long-term period (during the first five years) and scenarios 3 to 5 depend on the short-term period (during the first four years). The highest revenue (RE) and cost (CO) per year were determined for scenario 1 (12 860 kEUR (RE) and 7316 kEUR (CO)) and scenario 5 (9680 kEUR (RE) and 5989 kEUR (CO)). The lowest revenue and cost belong to scenario 2 (12 773 kEUR (RE) and 7182 kEUR (CO)) and scenario 4 (9396 kEUR (RE) and 5538 kEUR (CO)). The revenue and cost of scenario 3 amounted to 9507 kEUR and 5854 kEUR, respectively.

**Fig. 2:**
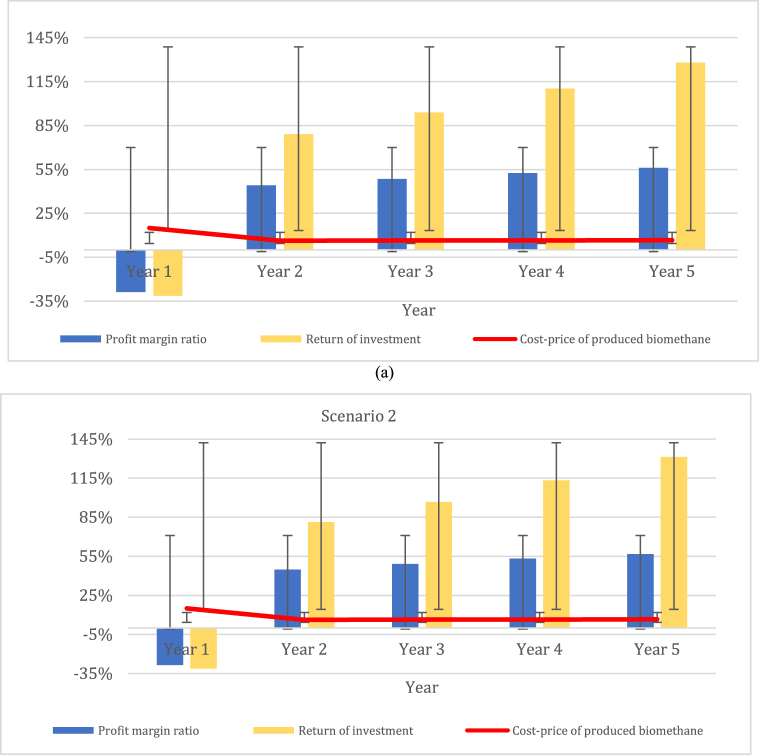
Assessment of the long-term perennial grass–legume mixture includes profit margin ratio, return of investment, and cost price for biomethane production: (a) scenario 1 and (b) scenario 2.

The financial benefit–cost ratio with subsidy shows that all scenarios have more than 1,0. This means that the benefits exceed the costs and a positive financial net present value is expected. Farmers could expect EUR 1.2 to 1.4 in benefits for each EUR 1 of costs. However, the financial benefit–cost ratio without subsidy shows that scenarios 1 and 2 are equal to 1,0. This means that, for farmers, EUR 1 in revenue is covered by EUR 1 of costs. In addition, scenarios 3 to 5 have less than 1. Hence, these projects should not be considered without subsidization. Fig. 2 shows the profit margin ratio, return on investment, and cost price for the scenario 1 and 2 biomethane production indicators for long-term perennial grass–legume mixtures during the first five years.

The indicator of profit margin ratio was estimated for scenario 1, ranging from −28,7 %–56.2 %, and for scenario 2, ranging from – 28.6 % to 56.8 %. The highest effect on return on investment can be seen in scenario 2, ranging from −31.3 % to 131.3 %, while the lowest effect can be seen in scenario 1, ranging from −31.3 % to 128.1 %. The lowest cost price was determined in the case of scenario 2, ranging from 14.9 % to 6.5 %, while the highest cost price was confirmed in the case of scenario 1, ranging from 15.1 % to 6.6 %. Scenario 2 offers more favorable conditions for profitability due to its broader range of potential returns on investment and lower production costs. However, both scenarios present important fluctuations and considerations, highlighting the importance of thorough analysis and management in optimizing profitability in biomethane production.

**Fig. 3:**
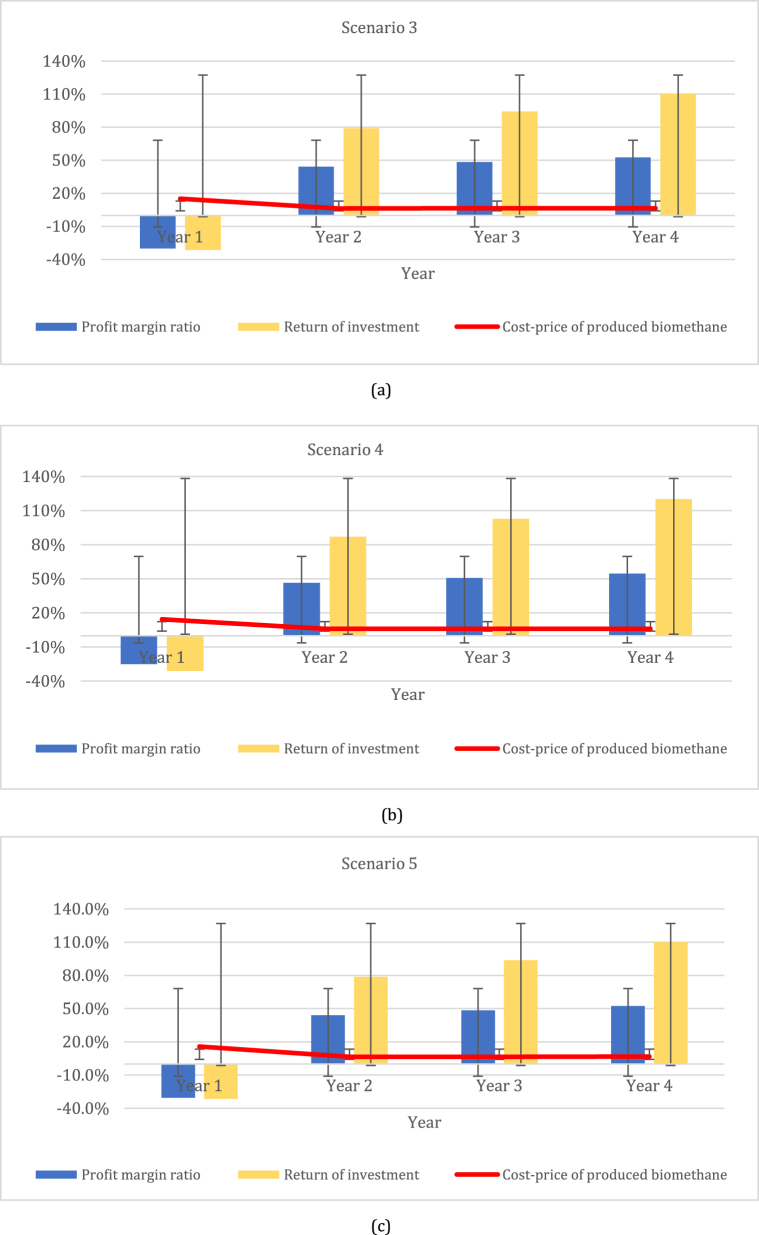
Assessment of the short-term perennial grass–legumes mixture, including profit margin ratio, return on investment, and cost price for biomethane production: (a) scenario 3; (b) scenario 4; and (c) scenario 5.

Fig. 3 shows the profit margin ratio, return on investment, and cost price in terms of biomethane production indicators for short-term perennial grass–legume mixtures during the first four-year period for scenarios 3 to 5.

The indicator of profit margin ratio for scenario 3 ranged from −30.0 % to 52.5 %; that for scenario 4 ranged from – 25.1 % to 54.6 %. The highest effect on return on investment was seen in scenario 4, ranging from −30.9 % to 120.2 %, while the lowest effect was found in scenarios 3 and 5, ranging from −31.4 % to 110.5 % and from −31.4 % to 110.0 %, respectively. The lowest cost price was determined in the case of scenario 4, ranging from 14.3 % to 6.1 %, while the highest cost price could be seen in case of scenario 3, ranging from 15.2 % to 6.5 %, and in scenario 5, ranging from 15.7 % to 6.6 %. Scenario 3 emerges as a potentially favorable option for profitability due to its lower cost price range, indicating the possibility of higher profitability. However, thorough analysis and management are crucial in navigating the fluctuations and complexities present in all scenarios in order to optimize profitability in biomethane production.

Based on our comparative analysis, scenarios 2 and 4 emerge as the most favorable options, exhibiting the highest profit margin ratio, return on investment, and the lowest cost price indicators. This suggests that these scenarios offer greater opportunities for profitability. In contrast, the other scenarios display lower profit margin ratios and returns on investment, along with higher cost price indicators. While these scenarios still hold profit potential, this is notably less than what is projected for scenarios 2 and 4. Additionally, it is worth noting that the first year of all perennial grass–legume mixtures indicated a negative effect, with costs surpassing the revenue earned. This underscores the initial investment required and the time needed for these activities to become financially viable. Table 8 shows the net present value and IRR indicators of perennial grass–legume mixtures. The net present value tells us whether the mixtures might positively affect biomethane production. The results show that all scenarios with subsidies had positive net present values. Scenario 2 is expected to have the most significant beneficial effect in relation to the NPV of scenario 1; in addition, scenario 4 might likely have the most significant beneficial effect in relation to the NPV of scenarios 3 and 5. The net present value without subsidy shows us that all perennial grass–legume mixture scenarios have a negative effect on investment for biomethane production. The highest negative effect was discovered in scenario 1, followed by scenario 3.

**Table 8:** Net present values for biomethane production in five perennial grass–legume mixture scenarios.

Scenarios	eNPV [EUR/yr]		IRR [%]	
	with subsidy	without subsidy	with subsidy	without subsidy
*Year 1 to year 5 time period*				
Scenario 1	1 590 057	−2 409 943	10,2	−1,1
Scenario 2	1 648 354	−2 351 646	10,5	−1,0
*Year 1 to year 4 time period*				
Scenario 3	422 383	−3 577 617	7,0	−7,6
Scenario 4	641 354	−3 358 646	8,2	−7,1
Scenario 5	439 416	−3 560 584	7,1	−7,4

In addition, the IRR results indicate that scenarios 2 and 4 with subsidies are two of the best means of biomethane production, accounting for 10,5 % and 8.2 %, respectively. Unfortunately, these same results indicate that all scenarios without subsidies might not result in profit.

**Fig. 4:**
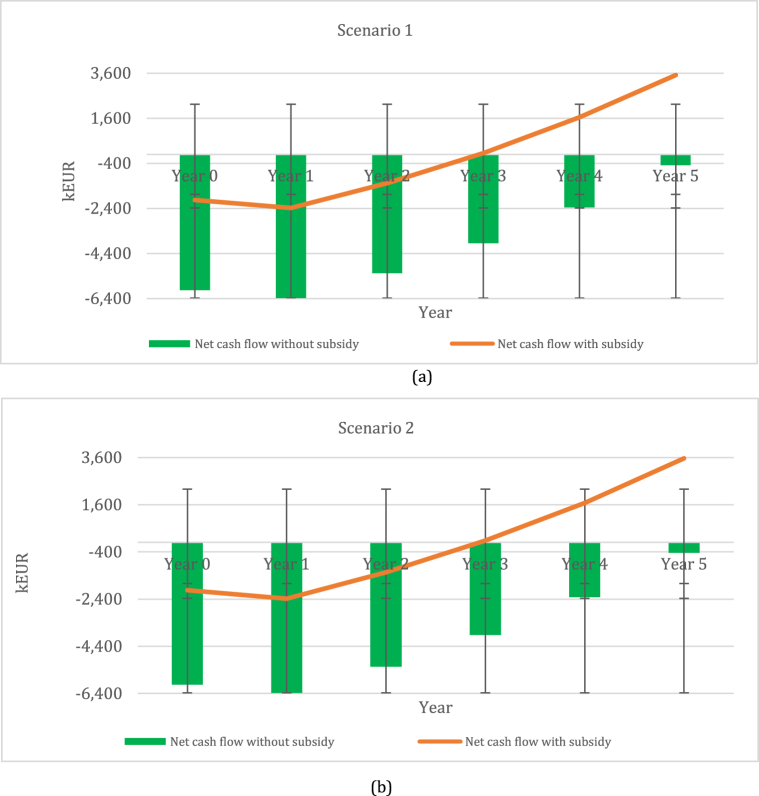
Net cash flow of long-term period perennial grass–legume mixtures: (a) scenario 1 and (b) scenario 2.

Fig. 4 represents the net cash flow indicator of perennial grass–legume mixtures during the first five-year period.

Scenarios 1 and 2 with subsidy show positive net cash flows in year 3, amounting to 54 kEUR and 82 kEUR, respectively. Scenario 2 has the highest expected net cash flow for biomethane production in year 5, accounting for 3561 kEUR, while scenario 1 has a lowest positive net cash flow in year 5, accounting for 3515 kEUR. The worst scenario tells us that net cash flow without subsidies would have a negative effect on biomethane production. The investment outflows should not exceeded the inflows. The biggest negative effect of net cash flow was determined in scenario 1, rather than scenario 2, ranging from 6029 kEUR to 485 kEUR and 6029 kEUR to 439 kEUR, respectively. Hence, it can be noticed that investment in perennial grass–legume mixtures without subsidies is not currently attractive and, due to this, more necessary resources should be allocated to financing biomethane production.

**Fig. 5:**
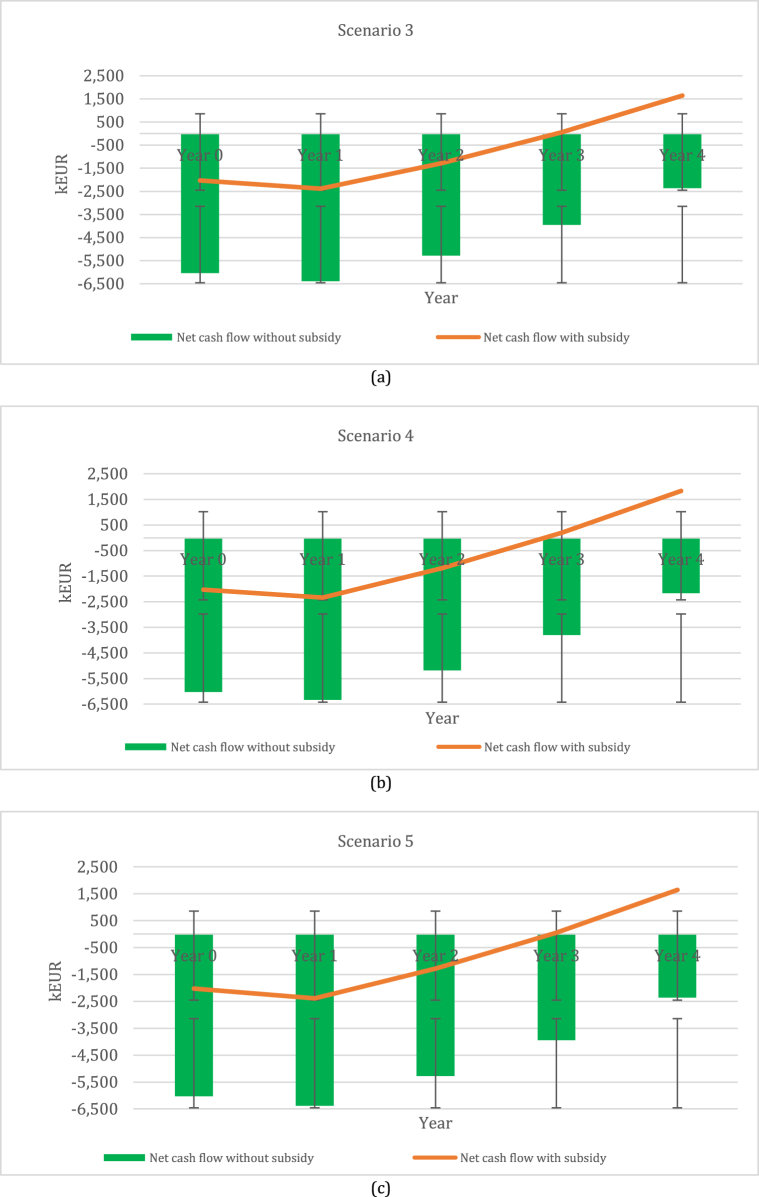
Net cash flow with and without subsidy for short-term period perennial grass–legume mixtures: (a) scenario 3; (b) scenario 4; and (c) scenario 5.

Fig. 5 represents the net cash flow indicator of perennial grass–legume mixtures during the first four-year period. Based on these results, it can be noted that all scenarios with subsidies have a positive net cash flow in year 3, amounting to 36 kEUR (scenario 3), 196 kEUR (scenario 4), and 52 kEUR (scenario 5). The best scenario was determined for scenario 4, accounting for 1830 kEUR in year 5, followed by scenario 5 at – 1661 kEUR and scenario 3 at −1624 kEUR. Furthermore, the indicator of net cash flow without subsidies in all scenarios represented a negative effect on biomethane production. The biggest negative effect was determined for scenario 3, ranging from 6029 kEUR to 2376 kEUR, followed by scenario 5, ranging from 6029 kEUR to 2339 kEUR. The least negative effect can be seen in scenario 4, ranging from 6029 kEUR to 2170 kEUR. Hence, these scenarios show that additional sources for biomethane production without subsidies should be sought.

As can be seen, the results emphasize the need to allocate more financial resources for biomethane production in order to promote this process effectively. According to Ranacher et al. (2021) [40], the economic feasibility of biogas investment projects is influenced by various factors. There is a need for mechanisms such as annual payments to incentivize farmer participation in projects, mitigate financial risks, and facilitate long-term investment in sustainable bioenergy systems. The results presented by Kintl et al. (2020) [14] evidence the importance of considering crop composition and its biochemical properties in optimizing biogas production efficiency. Their study highlights how the highest biogas yields were observed in maize combined with white sweet clover at specific ratios. In contrast, Cossel et al. (2021) [13] explore the potential of perennial crops for biogas production over long-term growth periods. While these crops may yield less biogas compared to maize or other perennials, like cup plant and Virginia mallow, their high lignin and low hemicellulose contents make them suitable for combustion. Grinwald et al. (2020) [41] explored perennial grass in combination with cup plants for more ecologically benign bioenergy production. They underscore the importance of factoring in biogas yield, as well as broader ecological and sustainability considerations, when evaluating bioenergy crop options. Additionally, Tilvikiene et al. (2021) [18] emphasize the importance of understanding the agronomic factors that affect feedstock yield and quality in enabling informed decision-making in feedstock selection and management practices. Møller and Martinsen (2013) [42] also underscore the importance of considering various input feedstocks. The different sizes of biogas plants shed light on the intricacies of feedstock composition and its impact on greenhouse gas (GHG) cost reduction. The variability in results depending on the type of plant material used indicates the complexity of optimizing biogas production for maximum environmental benefit. However, Bedana et al. (2022) [43] provide insights into the profitability of different sizes of biogas plants. Their findings underscore how medium-sized biogas plants provide the highest return on investment, indicating that profitability alone may not be the sole determinant of plant size selection. In contrast, Cucchiella et al. (2017) [44] emphasize that, while small plants show promise, profitability remains elusive in many cases, underscoring the challenges associated with economic viability in the absence of sufficient government support. Tonrangklang et al. (2022) [45] confirm the necessity of government funding for biomethane plants with capacities of less than 6 tons per day. Their findings underline the importance of supportive policy frameworks in facilitating the development and deployment of renewable energy technologies. The findings presented by Ahmed and Busadee (2023) [46] contribute to a nuanced understanding of feedstock selection and economic profitability in biogas production. Their study emphasizes the need to careful consider feedstock characteristics, market dynamics, and investment parameters in order to optimize economic returns and achieve long-term sustainability in biomethane production. It also confirms the results of the present study, in that government support is necessary to achieve economic efficiency and profitability in biomethane production. According to Pasini et al. (2019) [47], a significant aspect of the anaerobic digestion process is two systems for biomethane distribution, i.e., liquefied biomethane and biomethane grid injection. The insights provided by Oleszek et al. (2020) [16] revealed the importance of optimizing the fertilization process to achieve a balance between biomass productivity and energy efficiency in biogas production systems. Mel et al. (2015) [48] tested the anaerobic digestion of fruit and vegetable waste for biomethane production; the economic analysis showed that the profit margin ratio was achieved at 11 %, that the rate of return on investment model was at 12 %, and that the payback period was 8.2 years. These studies emphasize the complexity of optimizing biogas production while balancing productivity, environmental sustainability, and ecosystem services. Incorporating diverse crop compositions, considering their biochemical properties, and leveraging perennial crops offer promising pathways toward more sustainable and ecologically benign agricultural bioenergy production systems. Consequently, ongoing research endeavors are focused on identifying and refining the most effective plant combinations to maximize biomethane potential.

## 4 Conclusions

In this study, we carried out a comparative analysis of five perennial grass–legume mixture scenarios. Two scenarios were long-term (five years and beyond), and three scenarios were short-term (during the first four years). The main legume grass was red clover, and other, complex bell grasses, Timothy, P. Ryegrass and C. Cocksfoot, were included. Our comparative analysis included seven financial indicators, and all the indicators, except profit indicators, were analyzed from two perspectives: with and without subsidy.

The financial benefit ratio indicator revealed that all scenarios should be subsidized for biomethane production. Otherwise, the costs would not be able to cover the revenue. The profit indicators showed that the highest profit margin ratio and return on investment were determined for scenarios 2 and 4; these scenarios also saw the lowest cost price indicators. Profitability was also calculated for the other scenarios, but these presented less feasibility and higher risks. The indicator of net present value showed that the highest benefits could be seen in scenarios 2 and 4 with subsidy, while other scenarios had fewer benefits. The IRR result indicator also showed that scenarios 2 and 4 with subsidy are some of the best mixtures for biomethane production. However, all scenarios without subsidies presented no benefit and should not be promoted for biomethane production. The indicator of net cash flow revealed that the highest effect was determined by subsidization policies in scenarios 2 and 4, in which positive net cash flows might be reached in the third year. Unfortunately, the indicator of net cash flow without subsidization does not promise a positive effect, with outflow investment not exceeding inflow. Hence, all scenarios without subsidy are unattractive for biomethane production.

Our results emphasize the importance of government support in promoting biomethane production in Lithuania. They suggest that, without subsidies, farmers may not be sufficiently motivated to participate in this field. Our comparative analysis highlights specific perennial grass–legume mixtures as promising candidates for biomethane production, particularly when supported by a subsidy investment policy. This suggests that governmental financial support can play a crucial role in incentivizing and facilitating the adoption of sustainable energy practices. In terms of future research, we recommend expanding this analysis by considering additional types of grass–legume mixtures in order to further investigate biomethane production investment. This would provide a more comprehensive understanding of the potential options available and contribute to the advancement of biomethane production technologies and policies.

## Funding statement

This research did not receive any distinct funding grant from any organizations.

## Data availability statement

The data supporting this study's findings are available on request from the corresponding author.

## CRediT authorship contribution statement

**Rita Bužinskienė:** Writing – review & editing.

## Declaration of competing interest

The authors declare that they have no known competing financial interests or personal relationships that could have appeared to influence the work reported in this paper.
